# Cellular Analysis and Comparative Transcriptomics Reveal the Tolerance Mechanisms of *Candida tropicalis* Toward Phenol

**DOI:** 10.3389/fmicb.2020.00544

**Published:** 2020-04-15

**Authors:** Hanyu Wang, Qian Li, Yuanyuan Peng, Zhengyue Zhang, Xiaolin Kuang, Xiangdong Hu, Ellen Ayepa, Xuebing Han, Getachew Tafere Abrha, Quanju Xiang, Xiumei Yu, Ke Zhao, Likou Zou, Yunfu Gu, Xi Li, Xiaoying Li, Qiang Chen, Xiaoping Zhang, Beidong Liu, Menggen Ma

**Affiliations:** ^1^Institute of Resources and Geographic Information Technology, College of Resources, Sichuan Agricultural University, Chengdu, China; ^2^Department of Applied Microbiology, College of Resources, Sichuan Agricultural University, Chengdu, China; ^3^College of Landscape Architecture, Sichuan Agricultural University, Chengdu, China; ^4^School of Forestry and Life Science, Chongqing University of Arts and Sciences, Chongqing, China; ^5^Department of Chemistry and Molecular Biology, University of Gothenburg, Göteburg, Sweden; ^6^State Key Laboratory of Subtropical Silviculture, School of Forestry and Biotechnology, Zhejiang A&F University, Hangzhou, China

**Keywords:** *Candida tropicalis*, morphological observation, phenol, reactive oxygen species (ROS), tolerance mechanism, transcriptome

## Abstract

Phenol is a ubiquitous pollutant and can contaminate natural water resources. Hence, the removal of phenol from wastewater is of significant importance. A series of biological methods were used to remove phenol based on the natural ability of microorganisms to degrade phenol, but the tolerance mechanism of phenol-degraded strains to phenol are not very clear. Morphological observation on *Candida tropicalis* showed that phenol caused the reactive oxygen species (ROS) accumulation, damaging the mitochondrial and the endoplasmic reticulum. On the basis of transcriptome data and cell wall susceptibility analysis, it was found that *C. tropicalis* prevented phenol-caused cell damage through improvement of cell wall resistance, maintenance of high-fidelity DNA replication, intracellular protein homeostasis, organelle integrity, and kept the intracellular phenol concentration at a low level through cell-wall remodeling and removal of excess phenol via MDR/MXR transporters. The knowledge obtained will promote the genetic modification of yeast strains in general to tolerate the high concentrations of phenol and improve their efficiency of phenol degradation.

## Introduction

Phenol, one of the aromatic compounds, is composed of the hydroxyl group and the benzene ring and is applied as the main material for the production of pesticides, antiseptics, slimicides, and medicinal preparations (Mishra and Kumar, 2017) but contaminate the natural water resources. It was found that the inhalation, skin contact, and ingestion of phenol can lead to damages of central nervous system (CNS) disorders and the kidneys (Mahgoub et al., 2014; Mishra and Kumar, 2017). Hence, the removal of phenol from wastewater is quite important for environmental protection and for the human health. A series of physicochemical methods have been explored and applied to remove phenol from the wastewater (Pinto et al., 2005; Matjie and Engelbrecht, 2007; Busca et al., 2008). However, the energy consumption, high cost, hazardous byproducts production, and poor efficiency of these methods limited their widespread applications (Banerjee and Ghoshal, 2011). The natural ability of microorganisms to degrade phenol, and a few biological treatments of phenol are explored and found to be more efficient than physicochemical methods (Jiang et al., 2005; Banerjee and Ghoshal, 2011; Yoneda et al., 2016). It is a challenge for the wide applications of biological methods that these compounds are toxic to microbial cells, can prolong the lag phase, and reduce the phenol degradation efficiency (Heipieper et al., 1991). Additionally, phenol can penetrate the cellular membrane and cause the increased membrane permeability and decreased membrane lipid-to-protein ratios (Heipieper et al., 1991), and can cause dysfunction of organelles. The membrane disruption of mitochondria induced by phenol can induce the accumulation of excessive ROS (Klaunig et al., 2011), which interact with proteins, DNA, and lipids, and then result in damage of cytoskeleton, DNA mutagenesis, and the programmed cell death (Ibraheem and Ndimba, 2013). Hence, the phenol tolerance of microorganisms is vital to the effective degradation of phenol as well as screening and isolation of phenol-tolerant strains and deciphering of their tolerance mechanisms are immensely important.

Although recent studies found that chromatin remodeling, efflux of toxic compounds, and aggregation of lipopolysaccharides on the outer cell membrane could increase the resistance of microorganisms to phenolic aldehydes derived from lignocellulose pretreatment (Gu et al., 2015; Yi et al., 2015), a part of the above mechanisms might be associated with the aldehyde tolerance. Another study focused on *R. opacus* PD630 found that phenol tolerance mainly involved the import and degradation of extracellular phenol (Yoneda et al., 2016), but the understanding of the tolerance mechanisms, not degradation mechanisms, of strains to phenol is not very clear.

*C*. *tropicalis* can not only utilize a range of carbon sources but also produce a range of biological products, including bioethanol, xylitol, and long-chain dicarboxylic acids (Horitsu et al., 1992; Kurihara et al., 1992; Sampaio, 1999). Additionally, *C. tropicalis* can tolerate high concentrations of phenol, salts, heat, furfural, and acetic acid (Adav et al., 2007; Wang et al., 2015). The genome of *C. tropicalis* has been completely sequenced (Butler et al., 2009), enabling to explore the molecular mechanisms of *C. tropicalis* in different conditions and considered as one of the promising strains for deciphering the tolerance mechanisms of microorganisms to phenol. Previous studies on the tolerance mechanisms of *C. tropicalis* to phenol have been focused on the degradation of phenol by biodegradation (Jiang et al., 2005; Klaunig et al., 2011), but not the molecular and cellular mechanisms. In this study, pre-cultured cells of *C. tropicalis* strain SHC-03 were treated with phenol in order to explore the above mechanisms via fluorescence microscopy and comparative transcriptomics.

## Materials and Methods

### Yeast Growth Conditions and Reagents

*C. tropicalis* SHC-03, isolated from a winery in She Hong, was grown in YPD medium (w/v, 1% yeast extract, 2% peptone, and 2% glucose) and in YPD medium supplemented with 0.5, 1.0, 2.0, and 3.0 g/L phenol with 200 rpm shaking at 30°C. The initial cell count in the culture was adjusted to ∼1.0 × absorbance value (optical density at 600 nm wavelength, OD_600_). With non-phenol-treated culture as control, the pre-cultures were cultivated in YPD medium overnight, then harvested by centrifugation at 4,000 rpm for 3 min at 4°C, and inoculated into 50 mL flasks with phenol-added YPD medium. Aliquots of cells and supernatant were harvested for analysis at various time points from 0 to 72 h. Cell density (OD_600_) of the cultures was determined by using a UV-2802 spectrophotometer (Unico, NJ, United States). Media ingredients were purchased from Sigma-Aldrich (St. Louis, MO, United States) or Sangon Biotech (Shanghai, China).

### Determination of Phenol Degradation Rate

The concentration of residual phenol was determined by the 4-aminoantipyrine spectrophotometric method. The reaction among phenol, 4-aminoantipyrine and potassium ferricyanide will develop a red color under alkaline conditions which can be measured by reading the absorbance at 510 nm (OD_510_).

### qRT-PCR Assays

To confirm the accuracy of results from RNA-seq, the qRT-PCR assay of the isolated mRNA for RNA-seq were implemented on a Mastercycler^®^ EP Realplex system (Eppendorf, Hamburg, Germany), using the procedures reported previously (Anders and Huber, 2010). A FastQuant RT Kit (With gDNase) and a Real Master Mix (SYBR Green) Kit (Tiangen Biotech Co., Ltd.) were respectively exploited to synthesize the first-strand cDNA and quantitative PCR reactions. Before qRT-PCR reactions were carried out, a calibrated messenger RNA (mRNA) control mix, which was gifted by Z. Lewis Liu (Bioenergy Research, NCAUR-ARS, US Department of Agriculture, Peoria, IL, United States), was integrated into the reaction system as a reference. Using online software of primer3^1^, the primers of the selected genes were designed for qRT-PCR assay (Supplementary Table S1). In the qRT-PCR reactions, three biological replicates and three technical replicates were performed, and the acquired data was analyzed using the developed methods (Liu and Slininger, 2007).

### RNA-Seq and Analysis

After the pre-cultured cells of *C. tropicalis* SHC-03 were transferred into the YPD mediums with different concentration of phenol, the phenol-treated cells and the non-phenol-treated cells were obtained at 3 h for RNA-Seq. RNA-Seq was conducted by Biomarker Technology Co. Ltd. (Beijing, China) with Hiseq-PE150 (Illumina, Inc., San Diego, CA United States). Based on the sequence of *C. tropicalis* MYA-3404 as reference genome, we analyzed the raw data by the BMKCloud cloud server^2^. The gene expression levels were analyzed using fragments per kilobase of the transcript per million mapped (FPKM) method (Florea et al., 2013). Differential expression analysis of two samples with three biological replicates was performed using the DEGseq R package (Anders and Huber, 2010) in a threshold criterion of the value of | log_2_(fold change)| ≥ 1 (FDR < 0.05). The annotations of differentially expressed genes (DEGs) were performed by the GO (Ashburner et al., 2000) and KEGG (Kanehisa et al., 2004) databases. We performed the statistical tests to identify the DEGs enriched in different KEGG pathways using KOBAS software (Mao et al., 2005).

### Fluorescence Microscopy and Cellular Analysis

The fluorescence microscopy, an Axio Imager A2 microscope (Carl Zeiss AG, Oberkochen, Germany) equipped with DIC, GFP, Rhod and DAPI filter lens, and different dyes, including 2′,7′-dichlorofluorescein diacetate (DCFH-DA), diaminophenylindole (DAPI), Mito Tracker^TM^ Green FM, ER-Tracker^TM^ Red, and Yeast Vacuole Membrane Marker MDY-64, were used to observe the integrity of the cellular structures to evaluate the accumulation of ROS, nuclear chromatin disorganization, mitochondrial membrane damage, endoplasmic reticulum membrane damage, and vacuole membrane damage. The processing procedure is carried out according to the corresponding experimental instruction, respectively (Supplementary Material 2). Before the harvested cells were stained by various dyes, all the reagents and buffers have been preheated at 30°C. To ensure the accuracy of experiment results, at least 100 cells were examined on each bright-field image.

### Determination of Intracellular SOD, CTT, GPX, GLR Activity, and GSH Content

After the pre-cultured cells of *C. tropicalis* SHC-03 were transferred into the YPD mediums with different concentration of phenol, the phenol-treated cells and the non-phenol-treated cells were harvested at 3, 6, and 9 h for determination of intracellular SOD, GPX, CTT, GLR activity, and GSH content. The corresponding values was detected using SOD, GPX, CTT, GLR, and GSH assay kit purchased from Solarbio (Beijing, China) according to the manufacturer’s instructions, respectively.

### Cell Wall Susceptibility Analysis

To understand the structural changes of cell wall induced by phenol, we performed the lyticase-dependent susceptibility analysis (Teixeira et al., 2009). Lyticase, a β-1,3-glucanase from *Arthrobacter luteus*, was purchased from Sigma-Aldrich (St. Louis, MO, United States). 10^7^ cells, harvested from different mediums with or without phenol, were washed twice with ultrapure water and resuspended in 2.0 mL PBS pH 7.0. Sixty microliters of a 2 mgmL lyticase was mixed into the cell suspensions, the decrease in the OD_600_ of each cell suspension was detected from 0 to 4 h.

## Results

### Growth and Phenol Consumption in Phenol-Supplemented Medium

**FIGURE 1:**
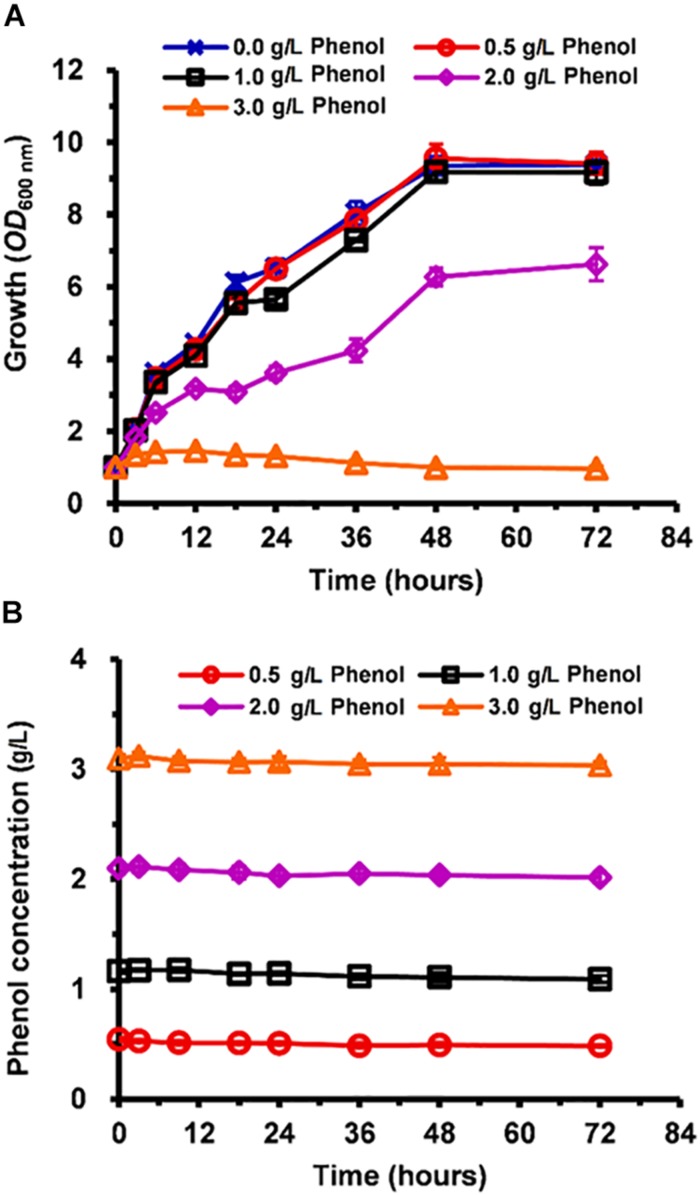
Inhibition of *C. tropicalis* SHC-03 cell growth by different concentrations of phenol. **(A)** Cell growth as measured by reading absorbance at 600 nm (OD_600_). **(B)** Phenol consumption.

Compared with the non-phenol-treated culture, the cell growth of phenol-treated *C. tropicalis* SHC-03 was slightly inhibited when the media contained 0.5 and 1.0 g/L phenol (Figure 1A). However, in the presence of 2.0 and 3.0 g/L phenol, cell growth was hindered by ∼40 and 100%, respectively (Figure 1A). The growth rate of cells treated with 2.0 g/L phenol remained at a low level from 3 to 24 h, implying that phenol influenced cell growth (Figure 1A). However, the cells returned to normal levels of growth after treatment for 24 h in the presence of 2.0 g/L phenol. The 4-aminoantipyrine spectrophotometric method revealed no significant change in the concentration of phenol in any of the treatments at 0–72 h (Figure 1B), which indicated that the phenol degradation mechanism of *C. tropicalis* SHC-03 was not activated in these conditions. To summarize, a range of tolerance mechanisms for protecting cells from the toxic damage of phenol were activated during the lag phase and RNA-seq technology and cytological techniques demonstrated the potential tolerance mechanisms of *C. tropicalis* to phenol.

### RNA-Seq, Transcriptomic Analysis, and qRT-PCR Assays

**FIGURE 2:**
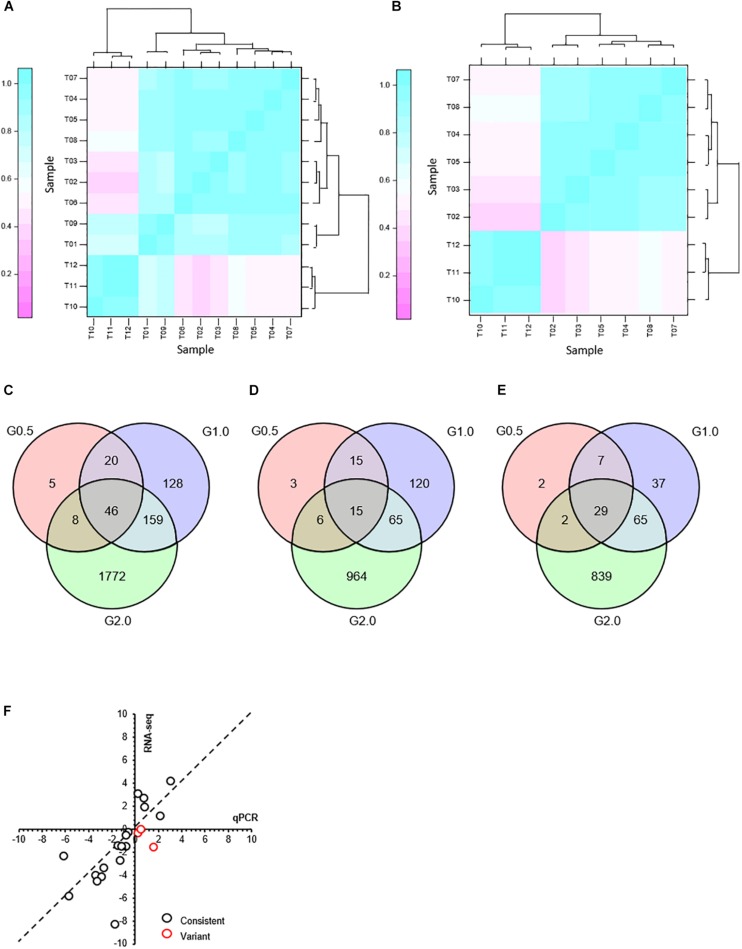
Transcriptome response to phenol after treatment for 3 h and comparison of expression levels of the selected genes as determined by RNA-seq and qRT-PCR. **(A)** Hierarchical cluster analysis of all replications. Replications of each phenol treatment group analyzed for RNA transcription are labeled as follows: 0.0 g/L phenol (T01, T02, T03); 0.5 g/L phenol (T04, T05, T06); 1.0 g/L phenol (T07, T08, T09); 2.0 g/L phenol (T10, T11, T12). Given the log_2_(fold change), expression levels of genes were clustered. **(B)** Hierarchical cluster analysis of the replications that did not include samples T01, T06, and T09. After T01, T06, and T09 were eliminated as outliers, three Venn diagrams were created based on differentially expressed genes in response to 0.5 g/L phenol (G0.5), 1.0 g/L phenol (G1.0), and 2.0 g/L phenol (G2.0), compared with the control group. **(C)** Differential expression of all genes. **(D)** Differential expression of up-regulated genes. **(E)** Differential expression of down-regulated genes. **(F)** Comparison of expression levels of the selected genes between the RNA-seq and qRT-PCR. The gene expression ratios of RNA-seq and qRT-PCR for 21 genes in response to 1.0 g/L phenol were calculated according to the values of log_2_(fold change) (treatment/control). The 21 selected genes included CTRG_00166, CTRG_00173, CTRG_00423, CTRG_00627, CTRG_00770, CTRG_01068, CTRG_01142, CTRG_01327, CTRG_01443, CTRG_01732, CTRG_01733, CTRG_01777, CTRG_02090, CTRG_02168, CTRG_02702, CTRG_03102, CTRG_03235, CTRG_03453, CTRG_03911, CTRG_03917, and CTRG_03930. The primers for RT-PCR of these selected genes are listed in Supplementary Table S1.

After incubation with or without phenol for 3 h, the cells were harvested for RNA-seq. Sequence reads data were archived at NCBI sequence read archive (SRA) with Accession Number PRJNA591802. The quality-control test results showed that the quality score (Q_30_), clean data, and sequencing depth of each sample were, respectively, more than 94%, 4 Gb, and 675× (Supplementary Table S2), which indicated a high level of accuracy for the RNA-seq results. To mitigate errors induced by biological variability between the samples, three biological replicates were used for the RNA-seq. The classification of the groups receiving different concentrations of phenol have been illustrated in Figure 2A, the correlations between T01, T06, T09 and their corresponding biological replicates were relatively low. To guarantee reliability and accuracy of the results from the differential expression analysis, T01, T06, and T09 were eliminated (Figure 2B).

The expression pattern of the 0.5 g/L phenol group was highly consistent with that of the control group; only 39 genes and 40 genes showed up- and down-regulated expression, respectively, with twofold changes in the 0.5 g/L phenol group compared with the control (Figures 2D,E). In contrast, for the 1.0 and 2.0 g/L phenol groups, 353 and 1,985 genes showed altered expression levels, respectively (Figure 2C). Among these genes, 215 and 1,050 genes were identified as up-regulated genes in the 1.0 and 2.0 g/L phenol groups, respectively (Figure 2D), and 138 and 935 genes, respectively, were repressed by phenol (Figure 2E).

In this study, 21 differentially expressed genes in response to 1.0 g/L phenol were selected for the accuracy of the results from RNA-seq through a qRT-PCR assay. The criterion for gene selection was a combination of high-low gene expression level (FPKM) and absolute value of log_2_(fold change). Through comparison and analysis, the expression levels of 18 of 21 genes (∼85%) were consistent in the trend of up- and down-regulation (Figure 2F). Of the other three genes, inconsistent with the data from RNA-seq, two displayed lower absolute values of log_2_(fold change) (Figure 2F). The results from RNA-Seq showed high accuracy, which contributed to the exploration of the phenol tolerance mechanism utilized by *C. tropicalis*.

### Accumulation and Scavenging of Reactive Oxygen Species (ROS) in Cells

**FIGURE 3:**
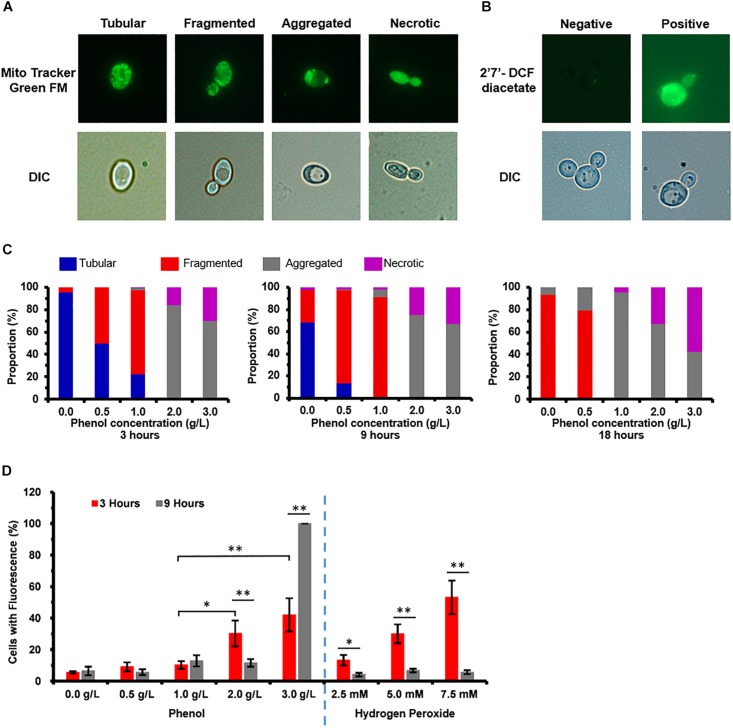
Mitochondrial membrane damage and accumulation of reactive oxygen species (ROS) caused by phenol. Representative images of cells stained with the mitochondria-specific dye Mito Tracker^TM^ Green FM and ROS indicator dye 2′,7′-dichlorofluorescein diacetate (DCFH-DA) are shown in the top column, and images taken using a differential interference contrast (DIC) lens are shown in the bottom column. **(A)** Morphological structure analysis of mitochondria. **(B)** Accumulation analysis of ROS. **(C)** Percentage of cells at each concentration of phenol that displayed tubular, fragmented, and aggregated mitochondria, as well as necrotic, at 3, 9, and 18 h. **(D)** Percentage of cells at each concentration of phenol and hydrogen peroxide that stained positive for ROS by DCFH-DA at 3 and 9 h, with cultures in medium containing 2.5, 5.0, and 7.5 mM hydrogen peroxide as positive controls for ROS. ^∗^*p* < 0.05; ^∗∗^*p* < 0.01 indicates significant differences. The data represent averages of three experiments. At least 100 cells were examined on each bright-field image.

In order of increasing damage, to phenol stress, mitochondrial membrane showed different types of morphologies these were: tubular, fragmented, aggregated shapes, and necrotic (Figure 3A). At 3 h, cells grown in media containing 0.0 and 0.5 g/L phenol displayed tubular and fragmented mitochondria, while cells grown in media containing 2.0 and 3.0 g/L phenol displayed aggregated and necrotic mitochondria (Figure 3C). In contrast, at 9 h, only 3% of the non-phenol-treated cells appeared to be necrotic. At the same time point, among cells grown in 0.5 g/L phenol, the percentage of cells with aggregated and necrotic mitochondria remained at a low level (Figure 3C). At 18 h, cells with tubular mitochondria were no longer seen in cells grown in media with or without phenol. In addition, compared with the results at 3 and 9 h, the distribution of cells containing aggregated mitochondria increased tremendously, with 95% of the yeast cells cultured in 1.0 g/L phenol showing aggregated mitochondria at 18 h. Furthermore, in cells cultured in 2.0 and 3.0 g/L phenol, the proportion of necrotic cells increased to 33 and 58% at 18 h, respectively (Figure 3C). In summary, the ratio of mitochondrial deformation increased with the elevation of phenol concentration and treatment duration.

As most of the exogenous ROS were produced by damaged mitochondria (Klaunig et al., 2011), ROS accumulation of the treated and untreated cells was detected. The percentage of cells staining positive for ROS was considered to be representative of severity of oxidative stress (Figure 3B). Cultures in media containing 2.5, 5.0, and 7.5 mM hydrogen peroxide served as the positive controls for ROS (Figure 3D). In the medium without phenol, at 3 and 9 h after treatment, 5.6 and 6.5% of the cells exhibited a positive ROS signal, respectively (Figure 3D). At 3 h, 9.1, 10.2, 30.3, and 42.1% of cells stained positive for ROS when 0.5, 1.0, 2.0, and 3.0 g/L phenol were present, respectively (Figure 3D), showing that the proportion of cells with ROS increased with increasing phenol concentration. At 9 h, the percent of cells staining positive for accumulated ROS was 5.7, 12.8, 11.6, and 100.0%, respectively (Figure 3D). The above results implied that the accumulation of ROS reached its peak 3 h after the treatment, while the accumulation of excessive ROS at this time might cause damage to DNA, proteins, and lipids (Gourlay and Ayscough, 2005; Perrone et al., 2008; Rowe et al., 2008), and that the intracellular ROS were eliminated by several molecular mechanisms between 3 and 9 h after the treatment.

Based on the transcriptome data, we found that the expression levels of most of these genes were not significantly up-regulated against 0.5, 1.0, and 2.0 g/L phenol (Supplementary Table S3). Among these genes, CTRG_04448, CTRG_04203, CTRG_01769, CTRG_00610, and CTRG_05111 exhibited 4.3-, 2.3-, 2.1-, 4.0-, and 3.2-fold down-regulation, and CTRG_03986, CTRG_00152, CTRG_06042, and CTRG_02189 displayed 3.0-, 2.5-, 2.1-, and 2.6-fold up-regulation when exposed to 2.0 g/L phenol (Supplementary Table S3). In addition, one of these genes, CTRG_00142, displayed 2.1-fold up-regulation when exposed to 1.0 g/L phenol. The transcriptome data demonstrated that the enzymatic antioxidant defense systems would not be significantly activated for scavenging excessive ROS at 3 h. In contrast, the above results from the determination of ROS showed that the excessive ROS in the phenol-treated cells were scavenged between 3 and 9 h (Figure 3D).

**FIGURE 4:**
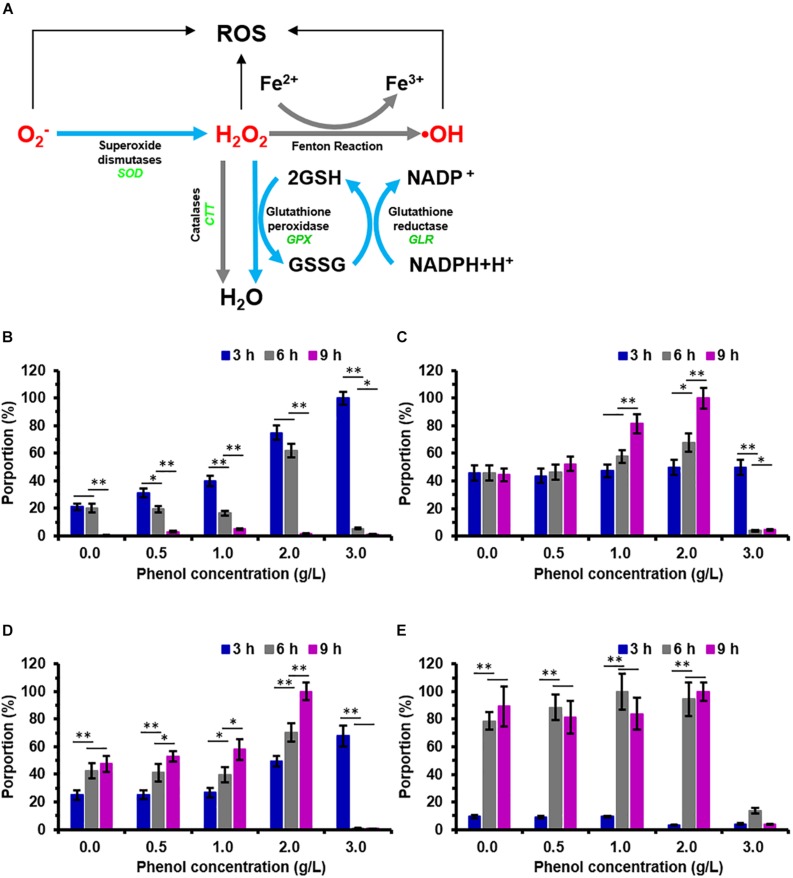
Pathway for reactive oxygen species (ROS) scavenging in *C. tropicalis* SHC-03 and the change in activity of ROS-related enzymes after treatment with phenol. **(A)** The pathway shows the scavenging of reactive oxygen species (ROS) catalyzed by enzymatic antioxidant defense systems containing SOD, GPX, CTT, GLR, and GSH. **(B–E)** Activities of **(B)** SOD, **(C)** GPX, **(D)** GLR, and **(E)** GSH after treatments with 0.0–3.0 g/L phenol for 3, 6, and 9 h. ^∗^*p* < 0.05; ^∗∗^*p* < 0.01 indicates significant differences.

The enzyme activity assays was carried out for SOD, CTT, and GPX in the phenol-treated and non-phenol-treated cells which showed that, at 3 h, the enzyme activity of intracellular SOD increased dramatically along with increasing phenol concentration (Figure 4B). The SOD activity of cells treated with 2.0 g/L phenol was significantly higher after 6 h than that of the cells treated with 0.0, 0.5, and 1.0 g/L phenol, but the SOD activity in cells treated with 3.0 g/L phenol was considerably lower (Figure 4B). The SOD activities of all the treatments were lower at 6 h than those of the corresponding treatments at 3 h. A few samples exhibited any SOD activity at 9 h (Figure 4B). Since GPX and CTT are the key enzymes for the reduction of H_2_O_2_, activities of both of the two enzymes were assayed. We found no significant differences in GPX activity in any of the treatments at 3 h. However, cells treated with 1.0 g/L phenol exhibited an increase in GPX activity at 9 h, and cells treated with 2.0 g/L phenol exhibited an increase in GPX activity at 6 and 9 h. At both 6 and 9 h, cells treated with 3.0 g/L phenol had lost their GPX activity almost completely (Figure 4C). These results indicated that GPX activity significantly increased in cells treated with 1.0 and 2.0 g/L phenol after 6 and 9 h, but that the high concentration of phenol (3.0 g/L phenol) caused the loss of cellular GPX activity at 6 h. The enzyme activity assays demonstrated no CTT activity in either phenol-treated or non-phenol-treated cells at the different processing times (data not shown). In addition, it has been found that high GLR activity and high GSH content can support high catalytic efficiency of GPX, which could protect the cells against ROS (Gill et al., 2013). In the present study, the enzyme activity of intracellular GLR was found to increase with phenol concentration at different processing times, except in those cells treated with 3.0 g/L phenol, at 6 and 9 h (Figure 4D). From the viewpoint of processing time, the cellular GLR activity after treatment for 9 h was higher than that after 3 and 6 h in the presence of 0.0–2.0 g/L phenol. However, no GLR activity could be detected in cells treated with 3.0 g/L phenol, at either 6 or 9 h (Figure 4D). The GSH content of cells treated with 2.0 and 3.0 g/L phenol was lower than that in cells treated with less than 2.0 g/L phenol at 3 h. However, after 3 h, the GSH content of cells treated with 0.0–2.0 g/L phenol rose rapidly after 6 and 9 h, and remained at a high level (Figure 4E). The GSH content remained at the same level in the presence of different concentrations of phenol after treatment for 6 and 9 h, except in the 3.0 g/L phenol group (Figure 4E).

### Damage to Chromatin and Protection of Chromosomal DNA

**FIGURE 5:**
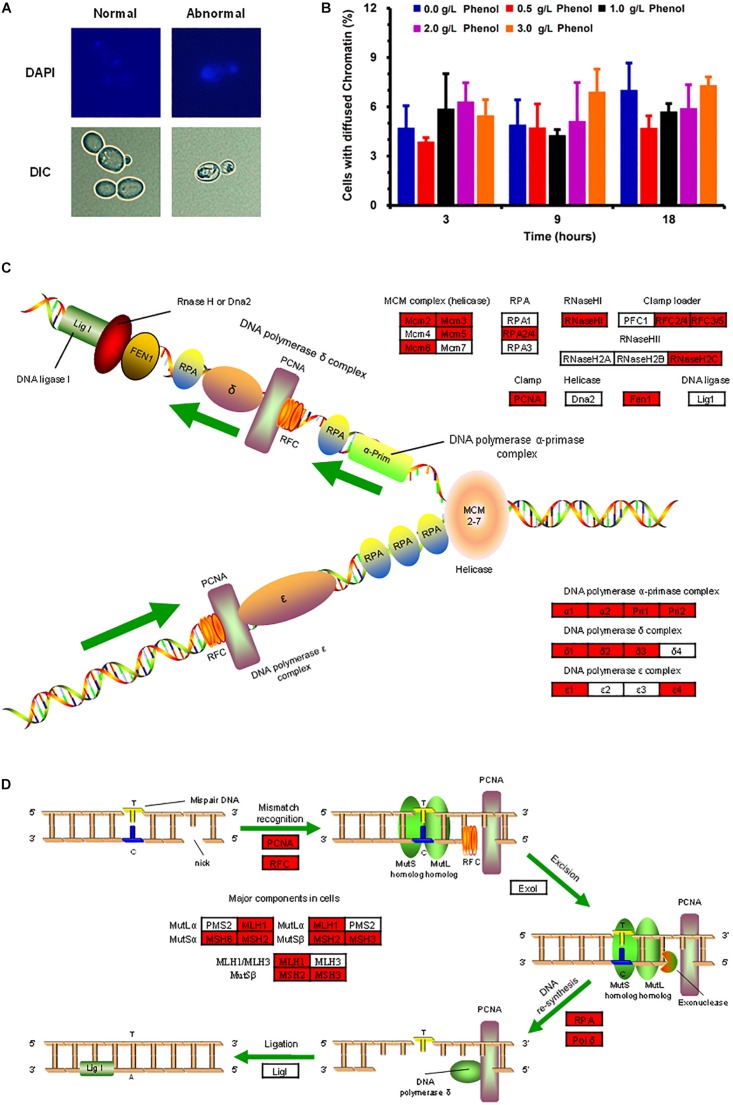
Morphological change in nuclear chromatin caused by phenol and the protection of nuclear DNA. **(A)** Representative images of cells stained with the DNA specific dye diaminophenylindole (DAPI) are shown in the top column, and images taken by a differential interference contrast (DIC) lens are shown in the bottom column. **(B)** Percentage of cells containing abnormal chromatin in the presence of each concentration of phenol at 3, 9, and 18 h. Data represent averages of three experiments. At least 100 cells were examined on each bright-field image. **(C,D)** DNA replication **(C)** and DNA mismatch repair **(D)** pathways. *Red* genes were up-regulated by 2-fold in the presence of 2.0 g/L phenol and listed in Supplementary Table S4.

The investigation on the chromatin damage caused by phenol showed that the structurally abnormal nuclear chromatin appeared larger and more diffuse, while the normal chromatin remained small and compacted (Figure 5A). The percentage of cells with abnormal diffuse nuclear chromatin was recorded in order to investigate the severity of nuclear chromatin damage. As illustrated in Figure 5B, the percentage of cells with nuclear chromatin disorganization remained at a low level (3.86–7.30%) when phenol was present or absent, which implied that 0.0–3.0 g/L phenol did not cause obvious damage to DNA and chromatin.

The transcriptome data indicated that most of the genes participating in DNA replication and DNA repair showed significant up-regulation in the presence of 2.0 g/L phenol (Figures 5C,D and Supplementary Table S4). Genes encoding ribonucleoside-diphosphate reductase subunit M2 (CTRG_01327 and CTRG_01698), as well as genes encoding ribonucleoside-diphosphate reductase subunit M1 (CTRG_01309), were highly expressed in cells exposed to 1.0 and 2.0 g/L phenol (Supplementary Table S4). The encoded proteins of the above genes are small and large subunits of ribonucleotide reductase (RNR), which plays a crucial role in dNTP production and DNA synthesis (Yao et al., 2003). Meanwhile, chromosome transmission fidelity protein 18 (CTRG_00975) was up-regulated by more than 4-fold in the presence of both 1.0 and 2.0 g/L phenol (Mayer et al., 2001; Supplementary Table S4).

### Accumulation and Degradation of Unfolded and Misfolded Proteins

**FIGURE 6:**
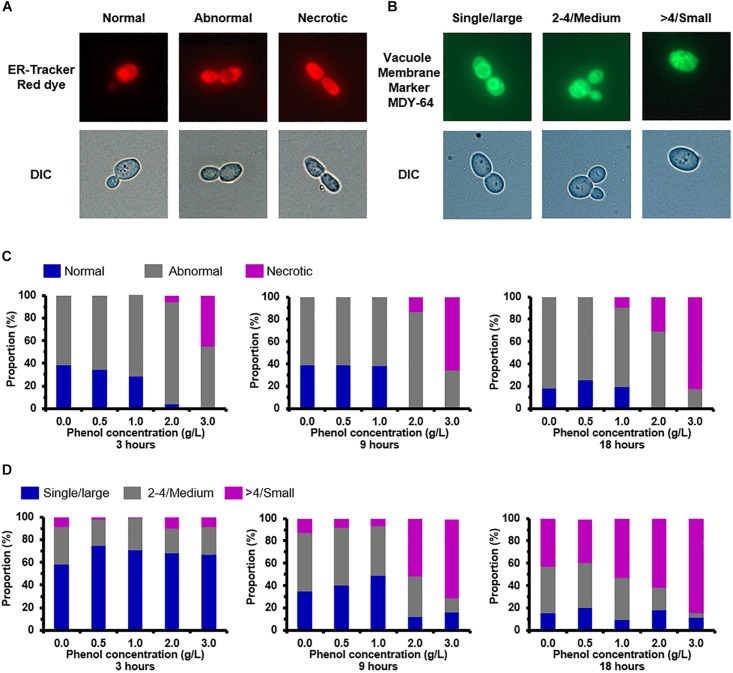
Endoplasmic reticulum (ER) and vacuole damage caused by phenol. Representative images of cells stained with the ER-specific dye ER-Tracker^TM^ Red and vacuole-targeted dye Yeast Vacuole Membrane Marker MDY-64 are shown in the top column, and images taken using a differential interference contrast (DIC) lens are shown in the bottom column. **(A)** Morphological structure analysis of the ER. **(B)** Morphological structure analysis of the vacuole. **(C)** Percentage of cells at each concentration of phenol that displayed normal, abnormal, and necrotic ER at 3, 9, and 18 h. **(D)** Percentage of cells at each concentration of phenol that contained large, medium, and small vacuoles at 3, 9, and 18 h. Data represent averages of three experiments. At least 100 cells were examined on each bright-field image.

The ER structures of the viable cells were divided into three groups: normal (unfolded), abnormal shapes (folded and fragmented), and necrotic (Figure 6A). When cells were exposed to 0.0, 0.5, and 1.0 g/L phenol, normal and abnormal ER structures were distributed roughly 35 and 65% of cells at 3 h, respectively (Figure 6C). Under the same conditions, there was no significant change in the ratio of cells containing normal and abnormal ER structures from 3 to 9 h, but there was a slight increase in the proportion of cells containing abnormal ER at 18 h (Figure 6C). The necrotic cells and the cells containing abnormal ER accounted for a large share of the observed cells treated with high concentrations of phenol (2.0 and 3.0 g/L) at various time points (Figure 6C). These results demonstrated that ER was not significantly injured by phenol at low concentrations (0.5 and 1.0 g/L), but was markedly damaged by phenol at high concentrations (2.0 and 3.0 g/L). In contrast, the recovery of cell growth in cells exposed to 2.0 g/L phenol after 24 h suggested that certain mechanisms provided enough protein to retain homeostasis in the cells. Previous studies have demonstrated that, after the unfolded protein response (UPR) and autophagy, cells could recover homeostasis and normal ER function (Senft and Ronai, 2015). HSPs functioning as chaperones were implicated in the reversal of amino acid oxidation and refolding of denatured proteins resulting from the UPR (Doong et al., 2003; Obeng et al., 2006; Lanneau et al., 2010). The expression of a gene CTRG_01443, annotated as “small heat shock protein 21” (Hsp21), was up-regulated by 11. 3-, 64-, and 13.9-fold change in the presence of 0.5, 1.0, and 2.0 g/L phenol, respectively (Supplementary Table S5). Besides, the gene CTRG_04372, annotated as co-chaperones in the Hsp70/Hsp90 family, was significantly up-regulated in the presence of 2.0 g/L phenol (Supplementary Table S5). Autophagy, as a conserved trafficking pathway, delivered unfolded or misfolded proteins, components, and organelles from the cytoplasm to the vacuole for degradation and recycling (Senft and Ronai, 2015). Four pivotal steps, including the activation of autophagy, the formation of the autophagosome, the cytoplasm-to-vacuole targeting (CVT) pathway, and vacuole fusion, ensured the effective degradation of the contents by vacuole hydrolases (Oku et al., 2006; Suzuki and Ohsumi, 2007; Senft and Ronai, 2015). In the presence of 2.0 g/L phenol, it was found that the expression of genes *IRE1*, *ATG11*, *ATG23*, and *ATG25*, related to the activation of autophagy and the formation of the autophagosome, were up-regulated by more than 2-fold (Supplementary Table S5). However, *APE1* and *AMS1*, which are related to autophagy, showed down-regulation by twofold (Supplementary Table S5).

The vacuole plays a key role in autophagy for the degradation and recycling of unfolded or misfolded proteins (Suzuki and Ohsumi, 2007) and accordingly changes of vacuole morphology in the non-phenol-treated and phenol-treated cells were observed. The observation revealed vacuoles in different configurations that could be classified as follows: a single large vacuole, two to four medium-sized vacuoles, and massively fragmented vacuoles (Figure 6B). After treatment for 3 h, compared with the other treatments, 75% of cells treated with 0.5 g/L phenol contained a single large vacuole. A single large vacuole was also predominant in cells treated with 1.0 and 2.0 g/L phenol (71 and 68%, respectively) (Figure 6D). Similarly processing time, cells treated with non-lethal doses of phenol (0.5, 1.0, and 2.0 g/L) exhibited a lower proportion of fragmented vacuoles than untreated cells (Figure 6D). At 9 h after treatment, cells treated with 1.0 g/L phenol (49%) showed the highest proportion of single large vacuoles, followed by cells treated with 0.5 g/L phenol (40%) (Figure 6D). From these observations, we speculated that low concentrations of phenol suppressed the fragmentation of the cellular vacuole. However, the cells treated with 2.0 and 3.0 g/L phenol exhibited a higher proportion of fragmented vacuoles than the untreated cells, after treatment for both 9 and 18 h (Figure 6D). Since mutation of *VAC8* has been correlated with vacuole fragmentation (Oku et al., 2006), the above results might be related to down-regulation of *VAC8* (Supplementary Table S5).

### Accumulation of Fatty Acid

**FIGURE 7:**
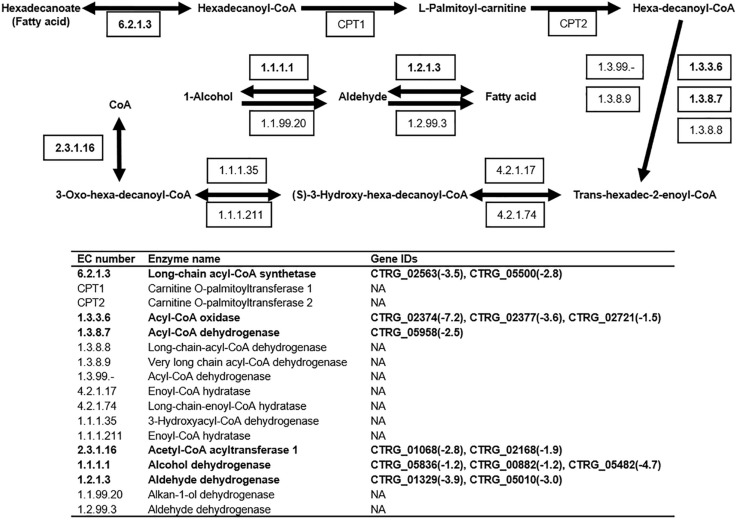
Fatty acid degradation pathway in *C. tropicalis* SHC-03. Known enzymatic reactions in *C. tropicalis* are shown in bold font. Expression levels of genes with values of log_2_(fold change) in response to 2.0 g/L phenol, compared with the control group, are shown. NA, none annotated.

The KEGG pathway enrichment analysis revealed down-regulation of 13 genes involved in fatty acid degradation (Figure 7). In this pathway, long-chain acyl-CoA synthetase (EC: 6.2.1.3) is responsible for the degradation of hexadecanoate (fatty acid), and the corresponding genes, CTRG_02563 and CTRG_05500, were down-regulated by 11.3- and 7-fold in response to 2.0 g/L phenol, respectively (Figure 7). When hexa-decanoyl-CoA was converted into trans-hexadec-2-enoyl-CoA, the expression of the corresponding genes (CTRG_02374, CTRG_02377, CTRG_02721, and CTRG_05958), annotated as acyl-CoA oxidase (EC: 1.3.3.6) and acyl-CoA dehydrogenase (EC: 1.3.8.7), showed 147-, 12.1-, 2.8-, and 5.7-fold decreases in cells treated with 2.0 g/L phenol, respectively (Figure 7). The genes CTRG_01068 and CTRG_02168, which encode acetyl-CoA acyltransferase1 (2.3.1.16), were 7- and 3.7-fold down-regulated in cells treated with 2.0 g/L phenol (Figure 7). In addition, the differential expression analysis implicated genes encoding aldehyde dehydrogenase (1.2.1.3) and alcohol dehydrogenase (1.1.1.1) in the conversion of fatty acid to alcohol (Figure 7). Specifically, CTRG_05836, CTRG_00882, CTRG_05482, CTRG_01329, and CTRG_05010 were significantly down-regulated in response to 2.0 g/L phenol exposure (Figure 7). These results suggested that decreased fatty acid degradation efficiency, caused by the down-regulation of expression of related genes, could elevate the intracellular fatty acid content or change the fatty acid component in cells (Kurosawa et al., 2015; Zhou et al., 2017).

### Cell-Wall Remodeling

**FIGURE 8:**
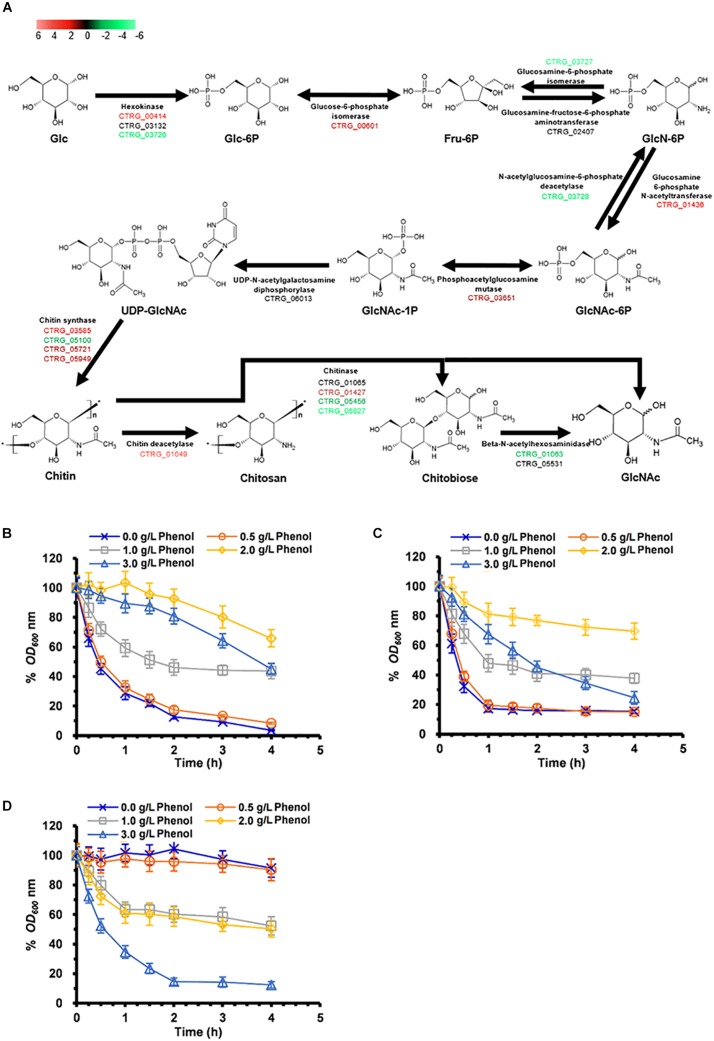
Synthesis and degradation of chitin in cells exposed to 2.0 g/L phenol and susceptibility to lyticase of cells treated with 0.0–3.0 g/L phenol for 3, 9, and 18 h. **(A)** Pathway showing genes involved in chitin synthesis and degradation. The color codes *red, black*, and *green* represent up-regulated, normal, and down-regulated expression, respectively, in the presence of 2.0 g/L phenol. Glc, D-Glucose; Glc-6P, D-Glucose-6-phosphate; Fru-6P, D-Fructose-6-phosphate; GlcN-6P, D-Glucosamine-6-phosphate; GlcNAc-6P, N-Acetyl-D-glucosamine 6-phosphate; GlcNAc-1P, N-Acetyl-alpha-D-glucosamine 1-phosphate; UDP-GlcNAc, UDP-N-acetyl-alpha-D-glucosamine; GlcNAc, N-Acetyl-D-glucosamine. The expression levels of the genes (log_2_ transformed) are listed in Supplementary Table S6. The mean values of relative optical density are presented with vertical error bars, each representing a single standard deviation (*n* = 3). **(B–D)** Change in cell density after treatment in PBS containing lyticase from 0 to 4 h after incubation. Cells treated for **(B)** 3 h, **(C)** 9 h, and **(D)** 18 h under phenol stress or no phenol stress. The mean values of relative optical density are presented with vertical error bars, each representing a single standard deviation (*n* = 3).

Previous studies have found that cell-wall remodeling can lead to increased ethanol resistance in yeast (Teixeira et al., 2014). Our comparative transcriptome analysis revealed >2-fold up-regulation of six genes related to cell-wall biogenesis and integrity in response to 1.0 and 2.0 g/L phenol, including CTRG_05721, CTRG_05949, CTRG_00608, CTRG_00036, CTRG_01855, and CTRG_03473 (Supplementary Table S6). Genes involved in cell-wall biogenesis may contribute to the increased resistance of *C. tropicalis* SHC-03 to phenol. A series of differential expression analyses of genes up-regulated in response to 2.0 g/L phenol revealed enrichment in the chitin synthesis pathway (Figure 8A and Supplementary Table S6). As illustrated in Figure 8A, during the conversion of glucose to chitin, the expression levels of CTRG_00414, CTRG_00601, CTRG_01436, CTRG_03651, CTRG_03585, CTRG_05721, and CTRG_05949 were markedly increased. In addition, most of the genes in the chitin degradation pathway (chitin to chitobiose or N-Acetyl-D-glucosamine), including CTRG_05456 and CTRG_05827 (encoding chitinase) and CTRG_01063 (encoding beta-N-acetylhexosaminidase), were significantly down-regulated in expression (Figure 8A and Supplementary Table S6). In the process of converting chitin to chitosan, CTRG_01049, which encodes chitin deacetylase, was dramatically up-regulated; by 26.5-fold (Figure 8A and Supplementary Table S6). The up- and down-regulated expression of these genes probably served to increase the accumulation of chitin and chitosan in the cell wall. To confirm the increased phenol tolerance of cells that had undergone cell wall remodeling, we conducted cell-wall susceptibility analyses of the phenol-treated and non-phenol-treated cells using lytic enzyme, a β-1,3-glucanase from *Arthrobacter luteus* (Teixeira et al., 2014). After 3 h, the cell density (OD_600_) of samples treated with 0.0 and 0.5 g/L phenol decreased significantly upon addition of lyticase to the medium. This decrease was even more pronounced in cells treated with 1.0 and 3.0 g/L phenol, decreasing to less than 10% of the initial cell density at 4 h (Figure 8B). The cell density of samples treated with 2.0 g/L phenol dropped slowly after lyticase was added into the medium for 4 h, decreasing to about 65% at 4 h (Figure 8B). After treatment with phenol for 9 h, the samples in 0.0 and 0.5 g/L phenol still displayed the most rapid cell-density decline in the lyticase-supplemented medium; the decrease in cell density in these samples was greater than the cell-density decrease seen in the samples in 1.0–3.0 g/L phenol (Figure 8C). However, the cell density of the sample in 3.0 g/L phenol dropped more quickly than all of the others, at 3 h (Figure 8C). The resistance of cells to lyticase after treatment with 1.0 and 2.0 g/L phenol for 9 h was similar to that of cells treated with the corresponding concentration of phenol for 3 h (Figure 8C). In cells exposed to phenol for 18 h, the cell density of the sample treated with 3.0 g/L phenol descended sharply, decreasing to about 12% after addition of lyticase to the medium and incubation for 4 h (Figure 8D). The cell density of samples treated with 1.0 and 2.0 g/L phenol for 18 h dropped slowly after the 4 h lyticase treatment. In contrast, the cell density in the samples with 0.0 and 0.5 g/L phenol showed no significant decline (Figure 8D). The results demonstrated that treatment for 3 and 9 h with 1.0–3.0 g/L phenol caused increased resistance of cells to lyticase, with a maximal effect seen at 2.0 g/L phenol. While 2.0 g/L phenol appeared to be the optimal concentration for lyticase resistance, cells treated with 0.0 and 0.5 g/L phenol also exhibited significantly increased resistance to lyticase after 18 h and cells treated with 3.0 g/L phenol for 18 h showed decreased resistance to lyticase compared with this treatment after 3 and 9 h.

### MDR/MXR Transport

One effective detoxification mechanism is the active efflux mechanism, which has been shown to reduce the level of intracellular toxic compounds, resulting in retention of the physiological activities of the cells (Dos Santos et al., 2014). The major facilitator superfamily (MFS) and ATP-binding cassette (ABC) subfamily are the two most important groups of multidrug/multixenobiotic resistance (MDR/MXR) transporters responsible for the efflux of toxic compounds (Sa-Correia et al., 2009). In response to at least two concentrations of phenol, the MFS genes CTRG_03938, CTRG_00385, and CTRG_03729 showed a >2-fold increase in expression (Supplementary Table S7). Specifically, CTRG_00385 was up-regulated by 7-, 18-, and 169-fold in response to 0.5, 1.0, and 2.0 g/L phenol. Additionally, a statistical analysis of differential gene expression showed that 14 genes belonging to the MFS and ATP-binding cassette (ABC) subfamily exhibited greatly up-regulated expression in the presence of phenol (Supplementary Table S7). As illustrated in Supplementary Table S7, 2, 6, and 10 transporter genes were significantly up-regulated in response to 0.5, 1.0, and 2.0 g/L phenol, which indicated that the number of up-regulated transporter genes increased with the rise in phenol concentration.

### Transcriptional Responses for Phenol Degradation

**FIGURE 9:**
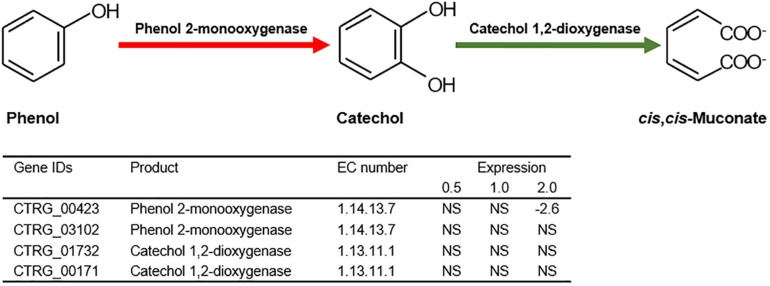
The first and second step of the β-ketoadipate pathway in the biodegradation of phenol. Expression levels of genes involved in the first and second step of the β-ketoadipate pathway are represented by the values of log_2_(fold change) in response to different concentrations of phenol, compared with the control group. NA, not significant.

Previous studies have illustrated that phenol degradation is the vital detoxification mechanism of *C. tropicalis* in response to phenol (Jiang et al., 2005; Varma and Gaikwad, 2009). Considering the molecular mechanism, the biodegradation of phenol mainly relies on subsequent enzymatic steps via the β-ketoadipate pathway in *C. tropicalis* (Krug et al., 1985). The crucial enzyme in this pathway, phenol 2-monooxygenase (EC 1.14.13.7), is responsible for the hydroxylation of phenol to catechol, which is a rate-limiting step (Krug et al., 1985; Figure 9). To date, two genes encoding phenol 2-monooxygenase, CTRG_00423 and CTRG_03102, have been discovered in *C. tropicalis* strains JH8 and MYA-3404, respectively (Butler et al., 2009; Long et al., 2014). In the present study, the comparative transcriptomics data did not indicate that CTRG_00423 and CTRG_03102 were significantly up-regulated under phenol stress after treatment for 3 h (Figure 9). In the second step of the β-ketoadipate pathway, catechol 1,2-dioxygenase (EC 1.13.11.1) is responsible for the conversion of catechol to *cis*,*cis*-muconate (Figure 9). CTRG_01732 and CTRG_00171, encoding catechol 1,2-dioxygenase, were not significantly up-regulated in response to different concentrations of phenol (Figure 9). The above results suggest that the phenol degradation mechanism of *C. tropicalis* SHC-03 was probably not activated.

## Discussion

In this study, the cells recovered and returned to normal growth 20 h after the treatment (Figure 1A), which indicated that detoxification and/or tolerance mechanisms should be activated to protect the cells from the toxicity of phenol and retain the viability of cells throughout the stress period. The determination of phenol concentration in each sample showed that the degradation of phenol in *C. tropicalis* was not activated (Figure 1B). Meanwhile, the transcriptome analysis of key genes (CTRG_00423, CTRG_03102, CTRG_01732, and CTRG_00171) related to phenol degradation demonstrated that phenol did not stimulate the phenol degradation mechanism of *C. tropicalis* in YPD medium (Figure 9). Therefore, we considered that the tolerance mechanism of *C. tropicalis* to phenol plays a critical role in maintaining the viability of phenol-treated cells.

In the present study, the treated cells exhibited serious damage to the mitochondrial membrane and high-level accumulation of ROS after the treatment for 3 h, which can cause damage to DNA, proteins, lipids, and the cytoskeleton (Figure 3; Gourlay and Ayscough, 2005; Perrone et al., 2008; Rowe et al., 2008), and then seriously affect the physiological and biochemical functions of cells. Interestingly, the percentages of the cells accumulating ROS declined to a lower level at 9 h (Figure 3D). Since efficient enzymatic and non-enzymatic antioxidant defense systems were found to be responsible for the scavenging of excessive ROS and protection of cells from oxidative damage (Hossain et al., 2006), we speculated that the associated genes, such as SOD, GPX, CTT, GLR, might be activated. The results from RNA-seq showed that these genes were not up-regulated in response to 3 h exposure to several concentrations of phenol (Supplementary Table S3), which implied that these antioxidant defense systems were not activated at the transcriptional level in response to phenol. To clarify the mechanism related to the scavenging of the intracellular ROS between 3 and 9 h, the activity assays of SOD, GPX, CTT, and GLR, as well as determination of GSH content, were conducted (Figure 4). As illustrated in Figure 4, the activities of SOD, GPX, and GLR showed obvious changes correlating with different conditions (phenol concentration and processing time). These changes were distinct from those at the transcriptional level. We hypothesized that differing regulation at the translational level might lead to the above inconsistent findings.

The results from the SOD activity assays showed that the SOD activities in different treatments remained high at 3 and 6 h, but were very low at 9 h, which implies that most of the O_2_^–^ probably had been converted to H_2_O_2_, and the SOD activity was sufficient to retain homeostasis of intracellular ROS at a low level after the treatment for 9 h (Figure 4A). During the conversion of H_2_O_2_ to H_2_O, GPX activities in the treatments with 1.0 and 2.0 g/L phenol exhibited a dramatic increase, but CTT activity was not detected in any of the treatments, implying that GPX played an important role in the reduction of H_2_O_2_ after the treatment for 6 and 9 h. Additionally, the high activity of GLR guarantees sufficient supplementation of GSH activity to contribute to the reduction of H_2_O_2_ catalyzed by GPX at 6 and 9 h (Figure 4D). In summary, the up-regulation of intracellular SOD activity (involved in the dismutation of O_2_^–^) at 3 and 6 h, and the up-regulation of intracellular GPX activity (involved in the reduction of H_2_O_2_) at 6 and 9 h, promoted the detoxification of endogenous ROS induced by phenol and low-level intracellular ROS.

The morphological structure analysis of nuclei demonstrated that phenol did not induce significant chromatin damage when the concentration of phenol was climbing (Figures 5A,B and Supplementary Table S4). Since chromatin protection mechanisms are associated with the DNA damage response (Masutomi et al., 2005), we speculated that ROS did not cause serious DNA damage. The transcriptome data showed that up-regulated expression of these genes, responsible for DNA repair, DNA synthesis, dNTP production, and chromatin protection, probably played a key role in protecting DNA, dNTPs, and chromatin from damage by ROS, and contributed to the maintenance of the viability of cells and the increased tolerance of *C. tropicalis* SHC-03 to phenol. Additionally, phenolic compounds (luteolin and quercetin) as antioxidants have been found to significantly decrease DNA damage (Lima et al., 2006). Perhaps phenol can function in a similar manner to luteolin or quercetin, helping cells to retain DNA and chromatin homeostasis.

Phenol-induced redox imbalance and ROS-induced protein damage can also cause unfolded or misfolded proteins to accumulate in the ER lumen and thereby induce ER stress (ERS) (Perrone et al., 2008; Senft and Ronai, 2015). The high concentration of phenol (2.0 g/L) caused serious damage to the ER at 3–18 h after treatment (Figure 6C), but the cell growth recovered in the presence of 2.0 g/L phenol. These results suggested that several pathways were triggered by ERS to guarantee the refolding of unfolded protein, the degradation of misfolded proteins, the restoration of normal ER function, and the maintenance of cell survival (Senft and Ronai, 2015). Two key fundamental pathways have been associated with response to ERS: one based on chaperone proteins and the other based on autophagy (Senft and Ronai, 2015). Previous studies have shown that HSPs can act as molecular chaperones associated with the folding, trafficking, protection, and renaturation of cellular proteins that have undergone the heat shock response (Walter and Buchner, 2002). Additionally, the up-regulation of the genes in the Hsp70/Hsp90 family is implicated in the adaptation and resistance of *C. albicans* to stress (Demand et al., 2001; Doong et al., 2003). In the present study, the genes, annotated as Hsp21and the co-chaperone of the Hsp70/Hsp90 family, were up-regulated by more than 2-fold in the presence of phenol (Supplementary Table S5). Hence, the accumulation of these HSPs might serves to protect the cellular proteins and facilitate the degradation of misfolded proteins via the ubiquitin proteasome system and relieve ERS, thereby improving phenol resistance in *C. tropicalis* SHC-03. Additionally, UPR can activate 27 autophagy-related genes (ATGs) which control autophagy in *S. cerevisiae* (Suzuki and Ohsumi, 2007). In the present study, the transcriptome data showed that the ATGs, except for *ATG11*, *ATG23*, and *ATG25*, were no marked changes in the expression level. In addition, expression of the hydrolase genes *APE1* and *AMS1*, the encoded proteins of which are specifically transported by autophagy and the CVT pathway (a type of selective autophagy) to the vacuole for hydrolyzation of proteins (Wang and Klionsky, 2003), was down-regulated at 3 h after the phenol treatment. We also surveyed the expression of genes belonging to the PERK-eIF2α pathway, *ATF6*, *IRE1*, *ATF4*, and *CHOP*, which have been associated with the induction and regulation of autophagy and chaperones (Senft and Ronai, 2015). Only *IRE1* (CTRG_04146) showed a change in expression, and its expression was up-regulated by more than 2-fold in the presence of 2.0 g/L phenol at 3 h. In summary, compared with autophagy, HSP-mediated proteasomal degradation played the more dominant role in the relief of ERS triggered by phenol at the early stage of treatment.

As a compartmentalized organelle, the vacuole contains a series of hydrolases which can degrade damaged or unfolded proteins from the cytoplasm in *S. cerevisiae* during autophagy (Wang and Klionsky, 2003). It has been found that increased vacuolar volume (or the fusion of a fragmented vacuole) can sufficiently improve the hydrolytic capacity for autophagy (Baba et al., 1994). Our morphological observation demonstrated that low concentrations of phenol might block the fragmentation of the cellular vacuole in order to allow the cell to retain efficient autophagy (Figure 6D). Unfortunately, the results from RNA-seq demonstrated that the expression of the genes *TORC1*, *SIT4*, and *VPS1*, which regulate the fragmentation or fusion of the vacuole, did not significantly change under phenol stress at 3 h (data not shown). Perhaps several unknown genes involved in vacuole fusion were responsible for the higher proportion of cells containing large vacuoles. Therefore, a follow-up study will be conducted to characterize these genes. However, we did find that the proportion of cells containing large vacuoles was lower in the cells treated with 2.0 g/L phenol than in the untreated cells, at 9 and 18 h (Figure 5D). As the knockout of *VAC8* (CTRG_04061) has been shown to cause fragmentation of vacuoles (Oku et al., 2006), our results can probably be attributed to significant down-regulation of *VAC8* (Supplementary Table S5). Since the increased synthesis of fatty acid could promote the formation of lipid membranes and increase the tolerance of *S. cerevisiae* and *Chlorella* strains to phenol (Schneiter et al., 2000; Yang et al., 2012; Zhou et al., 2017), the accumulation of fatty acid in this study was likely related to the increased tolerance of *C. tropicalis* to high concentrations of phenol. Interestingly, the accumulation of fatty acids was found to be associated with the fragmentation of the vacuole (Schneiter et al., 2000). This might explain how the proportion of cells containing fragmented vacuoles when exposed to under 2.0 g/L phenol stress was higher than that in the untreated samples at 9 and 18 h (Figure 6).

Since chitin is a structural microfibrillar component and is associated with cell wall rigidity (Bulik et al., 2003; Ruiz-Herrera et al., 2002), accumulation of chitin in cell walls in this study might lead to cell wall remodeling and then promotes the adaptation of cells to hypo-osmotic conditions, increases the resistance of cells to stress, reduces the production of intracellular ROS, and prevents the entrance of phenol into the cells (Aguilar-Uscanga and Francois, 2003; Deshpande et al., 1997). Additionally, the cell wall susceptibility analysis demonstrated that phenol certainly induced the increased resistance of cells to stress. However, it remains to be studied whether it was the accumulation of chitin induced by phenol, and related to cell wall remodeling, that improved the phenol resistance of *C. tropicalis*.

The active efflux mechanism can enable the cell to retain the intracellular drug/xenobiotic concentration at low levels and maintain the viability of cells under stress from these toxic compounds (Sa-Correia et al., 2009; Ma and Liu, 2010). In our study, at different concentrations of phenol, 14 genes encoding MDR/MXR transporters, especially CTRG_00385, showed significantly up-regulated expression (Supplementary Table S7). Additionally, as the phenol concentration increased, the up-regulated transporter genes became more numerous. Based on these results, we reasoned that there were definite connections between an active efflux mechanism and the tolerance and response of *C. tropicalis* to phenol. In *S. cerevisiae*, QDR1 (YIL120W), a multidrug transporter from the MFS, modulates the assembly of the outer spore wall and is responsible for resistance to quinidine, ketoconazole, and fluconazole (Nunes et al., 2001; Lin et al., 2013). Additionally, as illustrated in Figure 8A, CTRG_01049, which encodes chitin deacetylase, an enzyme that catalyzes the conversion of chitin to chitosan, was up-regulated by 26.5-fold. Since the spore wall is primarily composed of chitosan (Baker et al., 2007; Neiman, 2011), up-regulated expression of CTRG_01049 should lead to excess accumulation of chitosan in the spore wall. Since mature spores, as quiescent cells, exhibit resistance to organic solvents, heat, and digestive enzymes, and since chitosan and dityrosine layers of the spore wall prominently contribute to stress resistance, a hypothesis has been proposed that sporulation of *C. tropicalis* is activated in order to reduce the assault of phenol to cells (Pammer et al., 1992; Kupiec et al., 1997). However, according to eosin Y staining method (Lin et al., 2013), we observed no spores in the samples at 3, 9, and 18 h after cells were exposed to different concentrations of phenol (data not shown). This suggests the possible need for further exploration of the mechanisms underlying sporulation in *C. tropicalis*.

## Conclusion

**FIGURE 10:**
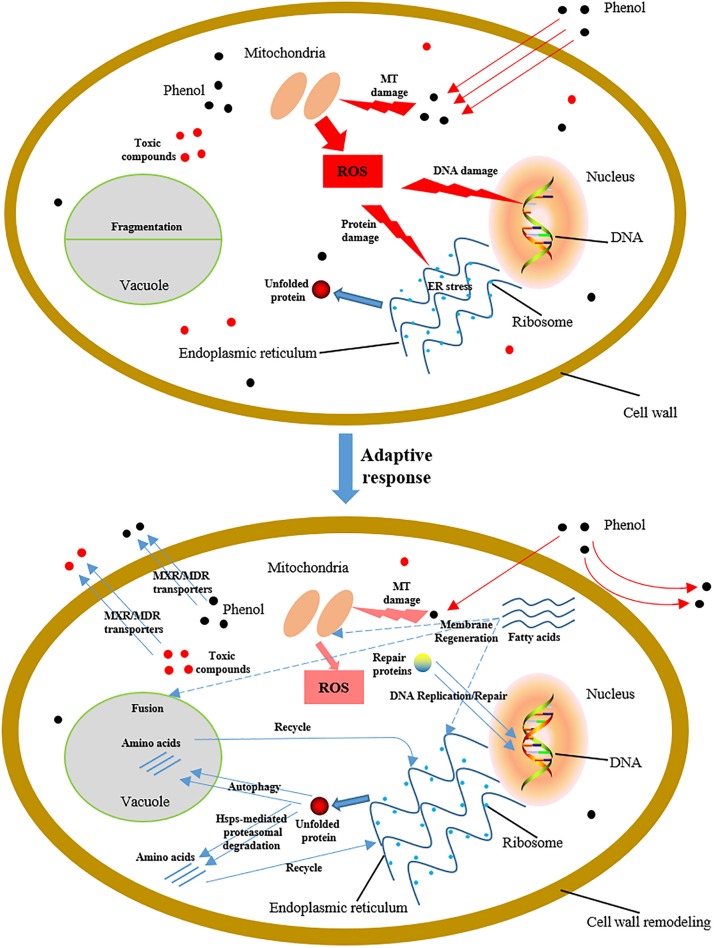
Schematic diagram showing cellular damage to *C. tropicalis* SHC-03 caused by phenol, and tolerance mechanisms to phenol at the early stage of treatment. MT, mitochondria.

*C. tropicalis* SHC-03 cells are treated with different concentrations of phenol as a representative of pollutants in environment for investigating the tolerance mechanisms of *C. tropicalis* to phenol. The experimental results indicated that phenol can result in massive accumulation of ROS. Although these intracellular ROS were found to be scavenged by SOD and GPX, the accumulation of ROS in the early stage after phenol treatment could cause damage to DNA, proteins, and lipids (Figure 10), and this stress could lead to morphological changes in the structure of the endoplasmic reticulum and vacuole. To reduce this damage, a series of protection mechanisms were activated. The DNA repair and synthesis system serve to ensure the stability of heredity, and key enzymes retain the fidelity of chromosome transmission and the stability of chromosomal structure. HSP-mediated proteasomal degradation and autophagy served to maintain intracellular protein homeostasis and accelerated recycling of proteins. Additionally, the accumulation of fatty acids contributed to the regeneration and repair of organelles with membrane systems. Cell wall remodeling minimized both import of phenol into the cells and production of intracellular ROS. The MXR/MDR transporters facilitated export of phenol and cytotoxic compounds from the cells, and maintained the intracellular drug concentration at low levels in order to maintain cell viability. This is the first study to shed light on the influence of phenol, a ubiquitous pollutant in wastewater, on the physiological characteristics of *C. tropicalis* SHC-03 and explore its tolerance mechanism to phenol stress.

## Data Availability Statement

The datasets generated for this study can be found in the Sequence files for all the treatment samples used in this study have been deposited at NCBI SRA with accession: PRJNA591802.

## Author Contributions

MM, HW, QL, QC, and XZ conceived and designed the project. QL, HW, YP, ZZ, XK, XHu, and XHa performed the experiments. QL, HW, QX, XY, KZ, LZ, YG, XiLi, and XiaLi performed the data analysis. HW, QL, YP, and ZZ wrote the manuscript. MM, BL, EA, and GA revised the manuscript. All authors read and approved the final manuscript.

## Conflict of Interest

The authors declare that the research was conducted in the absence of any commercial or financial relationships that could be construed as a potential conflict of interest.

## Footnotes

1 http://bioinfo.ut.ee/primer3-0.4.0/primer3/

2 http://www.biocloud.net
