# Supplementary material for: Cellular Analysis and Comparative Transcriptomics Reveal the Tolerance Mechanisms of Candida tropicalis Toward Phenol
Source: Front Microbiol. 2020 Apr 15;11:544. doi: 10.3389/fmicb.2020.00544 (PMC7179700; doi:10.3389/fmicb.2020.00544)
Supplement: Supplementary file 1 [file Table_1.DOCX]

**Table S1.** Primers used for qRT-PCR of the 21 selected genes

| Number | Gene | Primer ID | Forward primer (5'-3') |
| --- | --- | --- | --- |
| 1 | CTRG_00166 | qCTRG_00166_F | TCCAAATGGCCAGAGGTTAC |
|  |  | qCTRG_00166_R | TCCTCATCATGACCATCAAA |
| 2 | CTRG_00173 | qCTRG_00173_F | CATCTCCTTGGATAGGATGT |
|  |  | qCTRG_00173_R | GCCAACCCTTGATCATCTTC |
| 3 | CTRG_00423 | qCTRG_00423_F | CGTTTGAAAGTTGCTCGTGA |
|  |  | qCTRG_00423_R | ATCAAGATCAACCCCATCCA |
| 4 | CTRG_00627 | qCTRG_00627_F | TGGTGCCACTGCTCATAAAT |
|  |  | qCTRG_00627_R | GCCATAGCACTTGAACCAAT |
| 5 | CTRG_00770 | qCTRG_00770_F | CCATTAGGCGGATACTGGAA |
|  |  | qCTRG_00770_R | TGTTGCACCACATCCTCAAT |
| 6 | CTRG_01068 | qCTRG_01068_F | GCAGGTGTTCCACCAGAAAT |
|  |  | qCTRG_01068_R | AAAGACATTGGGCAGCAAAG |
| 7 | CTRG_01142 | qCTRG_01142_F | ACCGGTTTTCAAGGATGATG |
|  |  | qCTRG_01142_R | TTCTGCATTTGCTCTTGTGG |
| 8 | CTRG_01327 | qCTRG_01327_F | GCCTCCTTCTGGACTTGTGA |
|  |  | qCTRG_01327_R | GAAGCAGCGAAAAAGGCTAA |
| 9 | CTRG_01443 | qCTRG_01443_F | TTATGATTGTCGGGGTTGCT |
|  |  | qCTRG_01443_R | GCCAATGGACCAGCATACTT |
| 10 | CTRG_01732 | qCTRG_01732_F | TCCATACCCATGATGCTGAA |
|  |  | qCTRG_01732_R | CCTTGTCACAATGCCAAAGA |
| 11 | CTRG_01733 | qCTRG_01733_F | GGATTGCAAGTTTCCTCCAA |
|  |  | qCTRG_01733_R | ACTCTTGTGGCAGCTGGATT |
| 12 | CTRG_01777 | qCTRG_01777_F | TTATGATTGTCGGGGTTGCT |
|  |  | qCTRG_01777_R | GCCAATGGACCAGCATACTT |
| 13 | CTRG_02090 | qCTRG_02090_F | GCCATGTGGTCAGTATTTTG |
|  |  | qCTRG_02090_R | TGCTTTACCAGCAACTGCAA |
| 14 | CTRG_02168 | qCTRG_02168_F | GCTGGTGTTCCACCTGAAAT |
|  |  | qCTRG_02168_R | CCAGCAAAGGCTTCATTGAT |
| 15 | CTRG_02702 | qCTRG_02702_F | GGGCCAAGTACACTGATGGT |
|  |  | qCTRG_02702_R | TGGCACATGTCTTCATTGGT |
| 16 | CTRG_03102 | qCTRG_03102_F | GTGGCACCATCTCTTCACCT |
|  |  | qCTRG_03102_R | TGGTGTACGATCAGCCATGT |
| 17 | CTRG_03235 | qCTRG_03235_F | CACCCATTGTATTGCCATGT |
|  |  | qCTRG_03235_R | CACCATTCTCCGTCGGTATT |
| 18 | CTRG_03453 | qCTRG_03453_F | ACCTGAAGAAATTCCCAAGA |
|  |  | qCTRG_03453_R | TAACTCCTCCTCCCCGTTTC |
| 19 | CTRG_03911 | qCTRG_03911_F | TGGTATTTCTGCCACCAACA |
|  |  | qCTRG_03911_R | CCAATACAGCAGAAGCAGCA |
| 20 | CTRG_03917 | qCTRG_03917_F | AGACACATCATGGCCTACCC |
|  |  | qCTRG_03917_R | CAAACAACAGGGTCCCAATC |
| 21 | CTRG_03930 | qCTRG_03930_F | CAAGAGATTATCACCGTGGA |
|  |  | qCTRG_03930_R | CACAACGAAGGGCAAATTCT |

**Table S2.** The transcriptome sequencing data

| Sample | Clean reads (M) | Clean bases (Gb) | GC Content | %≥Q30 | Sequencing depth |
| --- | --- | --- | --- | --- | --- |
| T01 | 14.75 | 4.35 | 38.49% | 94.62% | 675 |
| T02 | 15.02 | 4.41 | 38.50% | 94.25% | 685 |
| T03 | 14.81 | 4.35 | 38.88% | 94.92% | 675 |
| T04 | 17.13 | 5.04 | 38.05% | 94.26% | 782 |
| T05 | 16.83 | 4.97 | 38.10% | 94.44% | 772 |
| T06 | 20.82 | 6.10 | 38.52% | 94.59% | 947 |
| T07 | 23.89 | 7.05 | 37.75% | 94.67% | 1,094 |
| T08 | 25.56 | 7.51 | 38.07% | 94.66% | 1,165 |
| T09 | 20.69 | 6.10 | 38.53% | 94.64% | 947 |
| T10 | 16.78 | 4.94 | 38.68% | 94.86% | 767 |
| T11 | 16.26 | 4.80 | 39.24% | 94.31% | 745 |
| T12 | 19.81 | 5.80 | 38.98% | 94.67% | 901 |

Sequencing depth=Clean bases/(Total number of genes×gene length). Total number of all the genes is 6441 in *Candida tropicalis* (<https://www.ncbi.nlm.nih.gov/genome>), and average length of all the genes in *C. tropicalis* is estimated to 1000 bp.

**Table S3.** Transcription levels of the key genes in antioxidant defense systems at 3 h after phenol treatment under different concentrations

| Gene IDs | Product | Category | EC number | Transcription levels | | |
| --- | --- | --- | --- | --- | --- | --- |
|  |  |  |  | 0.5 | 1.0 | 2.0 |
| CTRG_00159 | Superoxide dismutase | SOD | 1.15.1.1 | NS | NS | NS |
| CTRG_06027 | Superoxide dismutase | SOD | 1.15.1.1 | NS | NS | NS |
| CTRG_02366 | Superoxide dismutase | SOD | 1.15.1.1 | NS | NS | NS |
| CTRG_03952 | Superoxide dismutase | SOD | 1.15.1.1 | NS | NS | NS |
| CTRG_04448 | Superoxide dismutase | SOD | 1.15.1.1 | NS | NS | -2.1 |
| CTRG_04203 | Catalase | CTT | 1.11.1.6 | NS | NS | -1.2 |
| CTRG_03986 | Glutamate-cysteine ligase | GSH | 6.3.2.2 | NS | NS | 1.6 |
| CTRG_03089 | Glutathione synthase | GSH | 6.3.2.3 | NS | NS | NS |
| CTRG_00896 | Glutathionyl-hydroquinone reductase | GTO | 1.8.5.7 | NS | NS | NS |
| CTRG_02682 | Glutathione reductase | GLR | 1.8.1.7 | NS | NS | NS |
| CTRG_01769 | Hydroxyacylglutathione hydrolase | GLO | 3.1.2.6 | NS | NS | -1.1 |
| CTRG_00284 | Glutathione S-transferase | GST | 2.5.1.18 | NS | NS | NS |
| CTRG_00336 | Glutathione S-transferase | GST | 2.5.1.18 | NS | NS | NS |
| CTRG_00610 | Glutathione S-transferase | GST | 2.5.1.18 | NS | NS | -2.0 |
| CTRG_01617 | Glutathione S-transferase | GST | 2.5.1.18 | NS | NS | NS |
| CTRG_02273 | Glutathione S-transferase | GST | 2.5.1.18 | NS | NS | NS |
| CTRG_02466 | Glutathione S-transferase | GST | 2.5.1.18 | NS | NS | NS |
| CTRG_02946 | Glutathione peroxidase | GPX | 1.11.1.9 | NS | NS | NS |
| CTRG_02947 | Glutathione peroxidase | GPX | 1.11.1.9 | NS | NS | NS |
| CTRG_02948 | Glutathione peroxidase | GPX | 1.11.1.9 | NS | NS | NS |
| CTRG_02980 | Glutathione peroxidase | GPX | 1.11.1.9 | NS | NS | NS |
| CTRG_03995 | Glutathione peroxidase | GPX | 1.11.1.9 | NS | NS | NS |
| CTRG_00152 | Alkyl hydroperoxide reductase | AHP | 1.11.1.15 | NS | NS | 1.3 |
| CTRG_00142 | Alkyl hydroperoxide reductase | AHP | 1.11.1.15 | NS | 1.1 | NS |
| CTRG_05019 | Alkyl hydroperoxide reductase | AHP | 1.11.1.15 | NS | NS | NS |
| CTRG_02226 | Peroxiredoxin | PRX | 1.11.1.15 | NS | NS | NS |
| CTRG_02243 | Peroxiredoxin | PRX | 1.11.1.15 | NS | NS | NS |
| CTRG_04531 | Peroxiredoxin | PRX | 1.11.1.15 | NS | NS | NS |
| CTRG_05111 | Peroxiredoxin | PRX | 1.11.1.15 | NS | NS | -1.7 |
| CTRG_05987 | Thioredoxin reductase | TRR | 1.8.1.9 | NS | NS | NS |
| CTRG_05819 | Thioredoxin | TRX | -.-.-.- | NS | NS | NS |
| CTRG_04432 | Thioredoxin | TRX | -.-.-.- | NS | NS | NS |
| CTRG_06042 | Thioredoxin | TRX | -.-.-.- | NS | NS | 1.1 |
| CTRG_02189 | Thioredoxin | TRX | -.-.-.- | NS | NS | 1.4 |
| CTRG_05342 | Thioredoxin | TRX | -.-.-.- | NS | NS | NS |
| CTRG_01995 | Thioredoxin | TRX | -.-.-.- | NS | NS | NS |
| CTRG_04311 | Glutaredoxin | GRX | -.-.-.- | NS | NS | NS |
| CTRG_05073 | Glutaredoxin | GRX | -.-.-.- | NS | NS | NS |
| CTRG_05057 | Glutaredoxin | GRX | -.-.-.- | NS | NS | NS |
| CTRG_02680 | Glutaredoxin | GRX | -.-.-.- | NS | NS | NS |
| CTRG_04173 | Glutaredoxin | GRX | -.-.-.- | NS | NS | NS |

Transcription levels of the genes were represented by the values of log_2_(fold change) at 0.5 g/L phenol (0.5), 1.0 g/L phenol (1.0), and 2.0 g/L phenol (2.0) against the control (at 0.0 g/L phenol), respectively. *NS* Not significant.

**Table S4.** Transcription levels of the significantly up-regulated genes in DNA replication, mismatch repair and dNTP synthesis at 3 h after phenol treatment under different concentrations

| Gene IDs | Product | Name | EC number | Transcription levels | | |
| --- | --- | --- | --- | --- | --- | --- |
|  |  |  |  | 0.5 | 1.0 | 2.0 |
| CTRG_02317 | DNA polymerase alpha subunit A | α1 | 2.7.7.7 | NS | NS | 4.1 |
| CTRG_00716 | DNA replication licensing factor MCM2 | MCM2 | 3.6.4.12 | NS | NS | 2.1 |
| CTRG_01719 | DNA replication licensing factor MCM3 | MCM3 | 3.6.4.12 | NS | NS | 1.2 |
| CTRG_01667 | DNA replication licensing factor MCM5 | MCM5 | 3.6.4.12 | NS | NS | 1.7 |
| CTRG_00894 | DNA replication licensing factor MCM6 | MCM6 | 3.6.4.12 | NS | NS | 1.4 |
| CTRG_01746 | Replication factor A2 | RPA2 | -.-.-.- | NS | NS | 3.3 |
| CTRG_00214 | Proliferating cell nuclear antigen | PCNA | -.-.-.- | NS | NS | 3.1 |
| CTRG_04872 | Replication factor C subunit 2/4 | RFC2/4 | -.-.-.- | NS | NS | 4.0 |
| CTRG_01161 | Replication factor C subunit 3/5 | RFC3/5 | -.-.-.- | NS | NS | 1.8 |
| CTRG_02214 | Ribonuclease HI | RNaseH1 | 3.1.26.4 | NS | NS | 2.0 |
| CTRG_00332 | Ribonuclease H2 subunit C | RNaseH2C | -.-.-.- | NS | NS | 2.9 |
| CTRG_00417 | Flap endonuclease-1 | FEN1 | 3.-.-.- | NS | NS | 2.0 |
| CTRG_04155 | DNA polymerase alpha subunit B | α2 | -.-.-.- | NS | NS | 4.2 |
| CTRG_06141 | DNA primase small subunit | Pri1 | 2.7.7.102 | NS | NS | 2.0 |
| CTRG_03811 | DNA primase large subunit | Pri2 | -.-.-.- | NS | NS | 4.1 |
| CTRG_05098 | DNA polymerase delta subunit 1 | δ1 | 2.7.7.7 | NS | NS | 2.0 |
| CTRG_06077 | DNA polymerase delta subunit 2 | δ2 | -.-.-.- | NS | NS | 3.5 |
| CTRG_04830 | DNA polymerase delta subunit 3 | δ3 | -.-.-.- | NS | NS | 2.4 |
| CTRG_00708 | DNA polymerase epsilon subunit 1 | ε1 | 2.7.7.7 | NS | NS | 2.4 |
| CTRG_04576 | DNA polymerase epsilon subunit 4 | ε4 | 2.7.7.7 | NS | NS | 1.1 |
| CTRG_00069 | DNA mismatch repair protein MLH1 | MLH1 | -.-.-.- | NS | NS | 1.5 |
| CTRG_03762 | DNA mismatch repair protein MSH2 | MSH2 | -.-.-.- | NS | NS | 2.3 |
| CTRG_03593 | DNA mismatch repair protein MSH6 | MSH6 | -.-.-.- | NS | 2.3 | 3.8 |
| CTRG_01949 | DNA mismatch repair protein MSH3 | MSH3 | -.-.-.- | NS | NS | 1.5 |
| CTRG_01327 | RDR subunit M2 |  | 1.17.4.1 | 1.8 | 2.3 | 4.4 |
| CTRG_01309 | RDR subunit M1 |  | 1.17.4.1 | NS | 1.6 | 4.1 |
| CTRG_01698 | RDR subunit M2 |  | 1.17.4.1 | NS | 1.3 | 3.5 |
| CTRG_00975 | Chromosome transmission fidelity protein 18 | CTF18 | -.-.-.- | NS | 2.4 | 4.4 |

Transcription levels of the genes were represented by the values of log_2_(fold change) at 0.5 g/L phenol (0.5), 1.0 g/L phenol (1.0), and 2.0 g/L phenol (2.0) against the control (at 0.0 g/L phenol), respectively. *RDR* Ribonucleoside-diphosphate reductase. *NS* Not significant.

**Table S5.** Transcription levels of the key genes related to unfolded protein response and autophagy at 3 h after phenol treatment under different concentrations

| Gene IDs | Product | Name | EC number | Transcription levels | | |
| --- | --- | --- | --- | --- | --- | --- |
|  |  |  |  | 0.5 | 1.0 | 2.0 |
| CTRG_01443 | Small heat shock protein 21 | HSP21 | -.-.-.- | 3.5 | 6.0 | 3.8 |
| CTRG_04372 | Hsp70/Hsp90 co-chaperone | CNS1 | -.-.-.- | NS | NS | 1.6 |
| CTRG_04146 | Serine/threonine-protein kinase/endoribonuclease | IRE1 | 2.7.11.1 | NS | NS | 1.2 |
| CTRG_00216 | Autophagy-related protein 11 | ATG11 | -.-.-.- | NS | 4.7 | 5.6 |
| CTRG_00277 | Autophagy-related protein 23 | ATG23 | -.-.-.- | NS | NS | 1.8 |
| CTRG_01773 | Autophagy-related protein 25 | ATG25 | -.-.-.- | NS | NS | 1.7 |
| CTRG_05533 | Aminopeptidase I | APE1 | 3.4.11.22 | NS | NS | -2.0 |
| CTRG_00156 | Alpha-mannosidase | AMS1 | 3.2.1.24 | NS | NS | -2.6 |
| CTRG_04061 | Vacuolar protein 8 | VAC8 | -.-.-.- | NS | NS | -1.5 |

Transcription levels of the genes were represented by the values of log_2_(fold change) at 0.5 g/L phenol (0.5), 1.0 g/L phenol (1.0), and 2.0 g/L phenol (2.0) against the control (at 0.0 g/L phenol), respectively. *NS* Not significant.

**Table S6.** Transcription levels of the genes involved in the biosynthesis of cell wall in response to different concentrations of phenol at 3 h after treatment

| Gene IDs | Product | EC number | Transcription levels | | |
| --- | --- | --- | --- | --- | --- |
|  |  |  | 0.5 | 1.0 | 2.0 |
| CTRG_00608 | Biofilm and cell wall regulator 1 | -.-.-.- | 1.0 | 1.6 | 1.8 |
| CTRG_00036 | Cell wall integrity transcriptional regulator | -.-.-.- | NS | 1.1 | 2.8 |
| CTRG_01855 | Yeast-form wall Protein 1 | -.-.-.- | NS | 2.2 | 3.8 |
| CTRG_03473 | Chitin synthase regulatory factor 3 | -.-.-.- | NS | 1.5 | 1.6 |
| CTRG_00414 | Hexokinase | 2.7.1.1 | NS | NS | 2.8 |
| CTRG_03726 | Hexokinase | 2.7.1.1 | NS | -1.4 | -2.7 |
| CTRG_00601 | Glucose-6-phosphate isomerase | 5.3.1.9 | NS | NS | 1.6 |
| CTRG_03727 | Glucosamine-6-phosphate isomerase | 3.5.99.6 | NS | NS | -4.6 |
| CTRG_01436 | Glucosamine 6-phosphate N-acetyltransferase | 2.3.1.4 | NS | NS | 1.9 |
| CTRG_03728 | N-acetylglucosamine-6-phosphate deacetylase | 3.5.1.25 | NS | NS | -3.8 |
| CTRG_03651 | Phosphoacetylglucosamine mutase | 5.4.2.3 | NS | NS | 1.4 |
| CTRG_03585 | Chitin synthase | 2.4.1.16 | NS | NS | 1.9 |
| CTRG_05100 | Chitin synthase 1 | 2.4.1.16 | NS | NS | -1.7 |
| CTRG_05721 | Chitin synthase 2 | 2.4.1.16 | NS | 1.2 | 1.1 |
| CTRG_05949 | Chitin synthase 2 | 2.4.1.16 | NS | 1.4 | 1.3 |
| CTRG_01049 | Chitin deacetylase | 3.5.1.41 | NS | NS | 4.7 |
| CTRG_01427 | Chitinase | 3.2.1.14 | NS | NS | 1.2 |
| CTRG_05456 | Chitinase | 3.2.1.14 | NS | NS | -1.5 |
| CTRG_05827 | Chitinase | 3.2.1.14 | NS | NS | -4.0 |
| CTRG_01063 | Beta-N-acetylhexosaminidase | 3.2.1.52 | NS | NS | -1.8 |

Transcription levels of the genes were represented by the values of log_2_(fold change) at 0.5 g/L phenol (0.5), 1.0 g/L phenol (1.0), and 2.0 g/L phenol (2.0) against the control (at 0.0 g/L phenol), respectively. *NS* Not significant.

**Table S7.** Transcription levels of the significantly up-regulated transporter genes at 3 h after phenol treatment under different concentrations

| Gene IDs | Product | Transcription levels | | |
| --- | --- | --- | --- | --- |
|  |  | 0.5 | 1.0 | 2.0 |
| CTRG_00385 | MFS antiporter QDR1 | 2.8 | 4.2 | 7.4 |
| CTRG_03729 | Uncharacterized MFS-type transporter | 1.3 | 1.1 | -1.2 |
| CTRG_03938 | MFS transporter | NS | 1.2 | 1.3 |
| CTRG_03730 | Uncharacterized MFS-type transporter | NS | 1.9 | NS |
| CTRG_04584 | Uncharacterized MFS-type transporter | NS | 1.5 | NS |
| CTRG_05890 | Uncharacterized MFS-type transporter | NS | 2.0 | NS |
| CTRG_00077 | MFS transporter | NS | NS | 3.2 |
| CTRG_00952 | Uncharacterized MFS-type transporter | NS | NS | 3.0 |
| CTRG_01648 | MFS transporter | NS | NS | 3.2 |
| CTRG_01803 | ATP-binding cassette (ABC) subfamily | NS | NS | 1.7 |
| CTRG_01806 | MFS transporter | NS | NS | 1.7 |
| CTRG_04909 | Uncharacterized MFS-type transporter | NS | NS | 2.1 |
| CTRG_05714 | ATP-binding cassette (ABC) subfamily | NS | NS | 1.3 |
| CTRG_05999 | ATP-binding cassette (ABC) subfamily | NS | NS | 1.8 |

Transcription levels of the genes were represented by the values of log_2_(fold change) at 0.5 g/L phenol (0.5), 1.0 g/L phenol (1.0), and 2.0 g/L phenol (2.0) against the control (at 0.0 g/L phenol), respectively. *NS* Not significant.
