# Supplementary material for: Cellular Analysis and Comparative Transcriptomics Reveal the Tolerance Mechanisms of Candida tropicalis Toward Phenol
Source: Front Microbiol. 2020 Apr 15;11:544. doi: 10.3389/fmicb.2020.00544 (PMC7179700; doi:10.3389/fmicb.2020.00544)
Supplement: Supplementary file 2 [file Table_2.DOCX]

**Fluorescence microscopy and cellular analysis**

The fluorescence microscopy, an Axio Imager A2 microscope (Carl Zeiss AG, Oberkochen, Germany) equipped with DIC, GFP, Rhod and DAPI filter lens, was used to observe the integrity of the cellular structures to evaluate the accumulation of ROS, nuclear chromatin disorganization, mitochondrial membrane damage, endoplasmic reticulum membrane damage, and vacuole membrane damage. Before the harvested cells were stained by various dyes, all the reagents and buffers have been preheated at 30 °C. To ensure the accuracy of experiment results, at least 100 cells were examined on each bright-field image.

To detect the reactive oxygen species (ROS), 10 μg of 2’ 7’-dichlorofluorescein diacetates (DCFH-DA) (Sigma-35845) (using a 2.5 mg/mL stock in ethanol) was added into 10^7^ cells. After a 2-h incubation at 30 °C, cells were washed twice with ultrapure water, and resuspended in 0.1 mL phosphate buffered solution (PBS) pH 7.0 [43]. Cells was observed using the GFP filter lens.

To visualize DNA chromatin, 10^7^ cells were collected and washed twice with ultrapure water. To fix and stain the harvested cells, cells were resuspended by 1 mLof the mixture of ethanol and ultrapure water (v/v, 19:1), which had been blended with 1 μl of a 100 μg/mL diaminophenylindole (DAPI) (Sangon Biotech-E607303-0002). After resuspension, these suspensions were immediately centrifuged at 8,000 rpm for 1 min, and the depositions were washed twice with ultrapure water and resuspended in 0.1 mL PBS pH 7.0. Cells was observed using the DAPI filter lens.

To investigate the mitochondrial membranes of the phenol-treated cells, 10^7^ cells incubated with Mito Tracker^TM^ Green FM (Thermo Fisher Scientific-M7514) (100 nM) at 30 °C for 15-45 min. After staining, the cultures were centrifuged at 8,000 rpm for 1 min, and the supernatants were discarded. The collected cells were washed twice with ultrapure water, and resuspended in 0.1 mL PBS pH 7.0. Cells was observed using the GFP filter lens.

To monitor the morphological change of the ER induced by phenol stress, 1 μl of a 1 mM ER-Tracker^TM^ Red (Thermo Fisher Scientific-E34250) was added into 10^7^ cells, which had been washed twice with sterile deionized water and resuspended in 1.0 mL PBS pH 7.0. After incubation at 30 °C for 15-30 min, the stained cells were washed with sterile deionized water, and resuspended in 0.1 mL PBS pH 7.0. Cells was observed using the Rhod filter lens.

10^7^ cells were harvested and stained by Yeast Vacuole Membrane Marker MDY-64 (Thermo Fisher Scientific-Y7536) for visualizing the vacuole morphology. The cells were incubated at 30 °C for a maximum of 3-5 min. The cells were pelleted by centrifugation and resuspended in 0.1 mL PBS pH 7.0. Cells was observed using the GFP filter lens.
